# Role of Mitogen Activated Protein Kinase Signaling in Parkinson’s Disease

**DOI:** 10.3390/ijms19102973

**Published:** 2018-09-29

**Authors:** Anastasiia Bohush, Grazyna Niewiadomska, Anna Filipek

**Affiliations:** Nencki Institute of Experimental Biology, Polish Academy of Sciences, 3 Pasteur Street, 02-093 Warsaw, Poland; a.bohush@nencki.gov.pl (A.B.); g.niewiadomska@nencki.gov.pl (G.N.)

**Keywords:** apoptosis, ERK1/2, JNKs, mitochondrial dysfunction, neurodegeneration, neuro-inflammation, oxidative stress, p38 MAPKs, Parkinson’s disease

## Abstract

Parkinson’s disease (PD) is a neurodegenerative disorder caused by insufficient dopamine production due to the loss of 50% to 70% of dopaminergic neurons. A shortage of dopamine, which is predominantly produced by the dopaminergic neurons within the substantia nigra, causes clinical symptoms such as reduction of muscle mass, impaired body balance, akinesia, bradykinesia, tremors, postural instability, etc. Lastly, this can lead to a total loss of physical movement and death. Since no cure for PD has been developed up to now, researchers using cell cultures and animal models focus their work on searching for potential therapeutic targets in order to develop effective treatments. In recent years, genetic studies have prominently advocated for the role of improper protein phosphorylation caused by a dysfunction in kinases and/or phosphatases as an important player in progression and pathogenesis of PD. Thus, in this review, we focus on the role of selected MAP kinases such as JNKs, ERK1/2, and p38 MAP kinases in PD pathology.

## 1. Introduction

**Figure 1:**
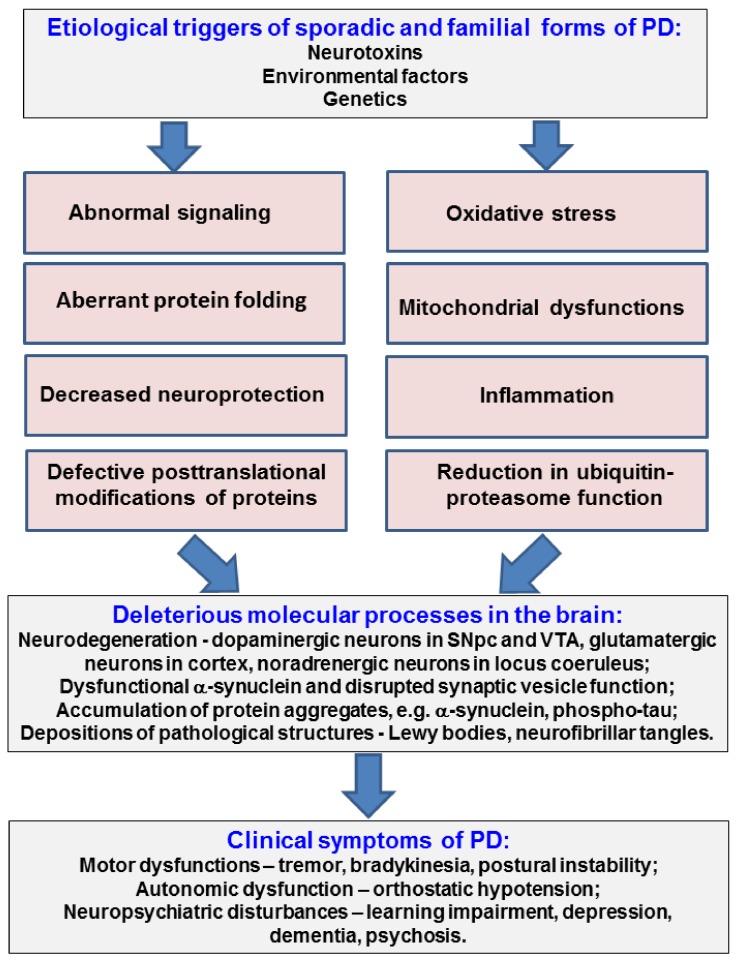
Principal pathological processes in PD etiology and clinical hallmarks of the disease. SNpc—substantia nigra pars compacta, VTA—ventral tegmental area.

Parkinson’s disease (PD) is a neurodegenerative disorder of the central nervous system (CNS), which affects about 1% of human population over the age of 60 [1] around the world and, for which, up to now, no cure has been developed [2]. Resting tremor, rigidity, hypokinesia, and postural instability are the four cardinal motor symptoms of PD resulting from the loss of dopaminergic neurons in the substantia nigra pars compacta, which is a key regulatory structure of basal ganglia circuitry. As the disease progresses, patients frequently develop cognitive impairment and depression. Most motor symptoms can be attributed to the degeneration of dopaminergic neurons within the substantia nigra pars compacta [3]. Nonetheless, in recent years, it has become increasingly appreciated that several other non-dopaminergic neuronal populations also degenerate (Figure 1). These include various autonomic nuclei and the locus coeruleus as well as glutamatergic neurons throughout the cerebral cortex. PD is characterized by the formation of specific inclusions called Lewy bodies (LBs) in neurons of several brain structures. LBs consist mostly of misfolded proteins such as α-synuclein, tubulin and microtubule associated proteins, ubiquitin, amyloid precursor protein, synaptic vesicle proteins, various enzymes, and chaperons/co-chaperons [4].

The MAP (mitogen-activated protein) kinase family is one of the oldest and evolutionally conserved family of serine/threonine protein kinases responsible for intracellular signaling in *Eukaryota* [5]. MAPKs (MAP kinases) regulate many physiological processes such as gene expression, mitosis, metabolism, cell differentiation and motility, stress response, survival, or cell death [6]. In mammalian cells, there are four main groups of conventional MAPKs: ERK1/2 (called also MAPK3 and MAPK1, respectively), ERK5, JNKs (JNK1, JNK2, and JNK3 called MAPK8, MAPK9, and MAPK10, respectively) and p38 MAPKs (p38α, p38β, p38γ, and p38δ called also MAPK14, MAPK11, MAPK12, and MAPK13, respectively). All these isoforms share sequence similarities but their cellular targets/substrates differ substantially. In addition, atypical MAPKs including NLK (Nemo-like kinase), ERK3/4, and ERK7/8 classified into a separate group have been described [6]. All these kinases collaborate in transmitting signals from numerous extracellular stimuli and control intracellular processes triggered by them. Thus, in consequence, MAPKs are capable of phosphorylating and altering the activities of countless substrates in different subcellular compartments. MAPK substrates have been found not only in the cytoplasm but also in mitochondria, the Golgi apparatus, the endoplasmic reticulum, and the nucleus [7,8].

### 1.1. MAPK Signalling 

The MAPK signalling cascade provides a mechanism for cells to respond to a catalogue of external signals. In fact, the diversity and specificity of cellular responses is facilitated through a linear cascade of events, which is comprised of a sequentially operating set of three evolutionarily conserved groups of protein kinases known as: MAPK, MAPK kinase (MAP2K), and MAPK kinase kinase (MAP3K). MAP3Ks are serine/threonine kinases, which are activated either via phosphorylation and/or due to the interaction with a small GTP-binding protein of the Ras/Rho family in response to extracellular stimulus. MAP3Ks activation results in phosphorylation and activation of MAP2Ks, which consequently stimulate MAPKs activity through dual phosphorylation of threonine and tyrosine residues positioned in the activation loop of kinase subdomain VIII. The activated MAPKs then phosphorylate target substrates specifically on serine or threonine residues followed by a proline residue. MAP2Ks such as MEK3 and MEK6 are activated by a wide range of MAP3Ks (MEKK1–3, MLK2/3, ASK1, Tpl2, TAK1, and TAO1/2), which become activated in response to oxidative stress, UV irradiation, hypoxia, ischemia, and cytokines including IL-1 (interleukin-1) and TNF-α (tumor necrosis factor alpha). Lastly, these events lead to altered gene expression and modulate crucial cellular functions under normal and pathological conditions such as Parkinson’s disease [9].

### 1.2. JNK Signaling 

JNKs (c-Jun N-terminal kinases) are a family of protein kinases activated in response to cytokines, growth factors, pathogens, and stress. JNK-mediated signaling pathways affect gene expression, neuronal plasticity, regeneration, apoptosis, or cellular senescence [10]. JNKs are activated through a dual phosphorylation of threonine and tyrosine residues within a threonine-proline-tyrosine (Thr-Pro-Tyr) motif by two MAP kinase kinases: MKK4 and MKK7. These two MAP kinase kinases can be inactivated by serine/threonine and tyrosine protein phosphatases [9]. In addition to the regulation by upstream kinases, the JNK signaling pathways are modulated by various scaffolding proteins including JNK-interacting protein 1, 2, and 3 (JIP1-3). The JNK family consists of 10 isoforms derived from three genes: JNK1 (four isoforms), JNK2 (four isoforms), and JNK3 (two isoforms). In mammalian cells, JNK1 and JNK2 are ubiquitously expressed while JNK3 is found mainly in the brain, heart, and testis [11]. In order to understand the biological function of JNKs, gene knockout studies were performed. It was found that mice deficient in JNK1, JNK2, JNK3, and JNK1/JNK3 or JNK2/JNK3 survived normally. Compound mutants lacking genes encoding JNK1 and JNK2 were embryonically lethal and had severe dysregulation of apoptosis of brain cells [12]. Under normal conditions, JNKs phosphorylate a variety of substrates. Examples of these substrates include a diverse assortment of nuclear transcription factors (Jun, ATF2, Myc, Elk1), cytoplasmic proteins involved in cytoskeleton regulation (DCX, Tau, WDR62), cell membrane receptors (e.g., BMPR2), mitochondrial proteins (e.g., Mcl1 and Bim), or proteins involved in vesicular transport (e.g., JIP1 and JIP3) [13].

In mammalian brains, JNK transcripts have been detected at levels similar to those in peripheral organs. However, JNK activity is noticeably higher in CNS than in peripheral organs. This activity can be increased by noninvasive environmental stimuli, which underlines the important role of JNKs in the brain [14] under norm and pathology and suggests that it may be implicated in neurodegenerative disorders such as Parkinson’s disease [15]. In this respect, it should be stressed that JNKs can be activated by a number of factors implicated in PD such as toxicants [16] and unfolded/misfolded proteins [17]. Some studies have demonstrated that JNKs are significantly activated in several common animal models of PD induced by neurotoxins such as MPTP (1-methyl-4-phenyl-1,2,3,6-tetrahydropyridine), 6-OHDA (6-hydroxydopamine) or LPS (lipopolysaccharide) [18,19,20,21]. Genetic deletion of JNK2 and JNK3 protects against MPTP-induced neurodegeneration in mice [22]. Moreover, some other studies have indicated that antioxidant and anti-inflammatory compounds provide neuroprotection in the MPTP and 6-OHDA model of PD, at least in part, through the inhibition of JNK activation [23,24]. In addition, it was found that inhibition of JNKs with the SP-600125 inhibitor protects dopaminergic neurons both from MPP+ (1-methyl-4-phenylpyridinium)-induced neuronal apoptosis in vitro and in MPTP and 6-OHDA models of PD [15]. Another inhibitor of JNKs, SR-3306, was found to reduce the loss of dopaminergic cell bodies in the substantia nigra and their terminals in the striatum [25]. SR-3306 was also shown to have a therapeutic effect in Alzheimer’s disease. A marked improvement of cognitive deficits, a significant decrease in the amount of β-amyloid plaques, and a decrease in tau phosphorylation in inflammatory responses were observed in transgenic animals treated for 12 weeks with the JNK inhibitor SP-600125 [26]. Recently, it has been reported that instant activation of JNK phosphorylation following treatment of cells with the HMGB1 (high mobility group box 1) protein cause an increase in the expression of tyrosine hydroxylase. The imbalance of this reduces dopamine synthesis and induces PD [27].

JNKs are not only implicated in the survival of dopaminergic neurons but also in dopamine transmission, which is, among the pathways, most impaired during the course of Parkinson’s disease [28]. Dopamine plays a central role in motor and cognitive functions as well as in reward processing by regulating glutamatergic inputs in the striatum. Release of dopamine rapidly exerts its influence on synaptic transmission and regulates both AMPA (α-amino-3-hydroxy-5-methyl-4-isoxazolepropionic acid) and NMDA (*N*-methyl-d-aspartate) receptors [29]. JNKs were found to be downstream targets of postsynaptic NMDA receptors and, moreover, NMDA activity is linked to the presence of a JNK scaffolding protein, JIP1. It was shown that NMDA-evoked glutamate release is controlled by presynaptic JNK-JIP1 interaction. Using JNK2 knock-out mice, it was proven that this kinase is essential in mediating glutamate release [30]. Activation of the glutamatergic pathway together with the dopaminergic one is responsible for synaptic plasticity, long-term potentiation (LTP), and long-term depression (LTD), which underlie motor learning. Accordingly, it has been found that LTP and LTD are altered in animal models of PD [31]. In addition, it has been found that the JNK1-Rac1 signaling pathway mediates phosphorylation of serine 295 in the PSD-95 (postsynaptic density protein 95) protein and, thus, enhances its synaptic accumulation and capability to recruit surface AMPA receptors and, lastly, potentiates excitatory postsynaptic currents [32]. It is worth mentioning that AMPA receptors, which are extremely relevant for synaptic plasticity, are physiological substrates of JNKs [33]. Lately, it has been found that, in a mouse model of PD, the JNK pathway is required for dopamine D1 receptor (D1R)-dependent modulation of corticostriatal synaptic plasticity. Pharmacological activation of D1R evokes a large increase in JNK phosphorylation. Electrophysiological experiments on brain slices from PD mice show that inhibition of JNK signaling in the pathway of striatal projection neurons prevents the increase in synaptic strength caused by activation of D1Rs [28].

It should be stressed that JNKs are implicated in other processes essential for neuronal homeostasis that seem to be severely dysregulated during Parkinson’s disease. For example, JNKs seem to play a role in protein transport in the brain. A study on *Caenorhabditis elegans* provides evidence that components of the JNK pathway are necessary for normal protein transport [34] and JNKs have been shown to modulate the interaction of kinesin with microtubules [35]. Therefore, inadequate JNK activity may be at the root of the impairment in the axonal transport frequently observed in PD and a number of many other neurodegenerative disorders [36,37].

JNK signaling is also linked to the apoptosis in neurons [38]. There is a study showing that, in cultured neurons, c-Jun activation is required for NGF (nerve growth factor) withdrawal-induced apoptosis and inhibition of c-Jun protects neurons from induced cell death. For instance, NGF deprivation-induced apoptosis is associated with the activation of the GTPase Cdc42 and JNKs in primary superior cervical ganglion sympathetic neurons. In addition, overexpression of the MAP3K apoptosis signal-regulated kinase 1 (ASK1), has been found to activate JNKs and to induce apoptosis in NGF-differentiated pheochromocytoma PC12 cells and primary rat sympathetic neurons [39]. On the other hand, inhibition of JNK signaling has been shown to reduce apoptosis of many other cells [40,41,42,43]. Therefore, JNKs may be critical for pathological cell death observed in Parkinson’s disease [44].

Numerous studies have implicated JNKs in oxidative stress, which is known to play an important role in Parkinson’s disease and other neurodegenerative disorders [45,46,47]. Research on *Drosophila melanogaster* showed that flies with mutations that accelerate JNK signaling accumulate less oxidative damage and live longer than wild-type flies [48]. JNKs activation has been also linked to stress evoked by misfolded proteins. Neuropathogenic forms of the huntingtin receptor and the androgen receptor were shown to inhibit axonal transport [49] and subsequent studies showed that this inhibition is mediated by JNK [37].

It should be noted that several other studies provide new insights into the role of JNK-mediated pathways in the control of the balance of autophagy in response to genotoxic stress i.e., the process that plays an important role in neurodegeneration including Parkinson’s disease [50,51,52].

### 1.3. ERK1/2 Signaling

The ERK1/2 (extracellular signal–regulated kinases 1 and 2) signaling cascade is a central MAPK pathway that plays a role in the regulation of various cellular processes such as proliferation, differentiation, development, learning, survival, apoptosis etc. [6]. This pathway is activated by growth factors [53], insulin [54], ligands of G protein-coupled receptors [55], or stress factors [56]. All these activators trigger a signal transmission by interacting with specific receptors such as receptors with tyrosine kinase activity (RTKs) or G protein-coupled receptors (GPCRs) [57].

Under a normal condition, the ERK1/2 pathway plays an essential role in the regulation of transcription. It phosphorylates and activates different transcription factors such as Elk1, c-Fos, p53, Ets1/2, c-Myc, and NFAT, which, in turn, activate numerous genes that encode proteins involved in proliferation [58]. Activation of ERK1/2 is crucial for efficient G_1_/S phase progression in a normal cell cycle. As mentioned above, ERK1/2 directly phosphorylate Elk1, which is involved in the expression of immediate-early genes. In addition, through direct phosphorylation of c-Fos, ERK1/2 promotes its association with c-Jun and the formation of a transcriptionally active AP-1 (activator protein 1) complex. The expression of cyclin D1, which is a protein that interacts with CDKs (cyclin-dependent kinases), permits G_1_/S transition depending on AP-1 activity [9]. In addition, ERK1/2 extend the MAPK cascade by phosphorylating and activating MAPKAPK (MAPK activated protein kinase) family members including RSKs (ribosomal S6 kinases), MSKs (mitogen and stress activated protein kinases), and MNKs (MAPK interacting protein kinases) [8]. Additionally, ERK1/2 phosphorylate members of the STAT transcription factor family (STAT1, STAT3, STAT4, and STAT5), which are known to mediate many aspects of survival, proliferation, differentiation [59], cytokine dependent inflammation [60], and apoptosis [61]. Genetic studies highlight differences between ERK1 and ERK2 isoforms. Some of them show that ERK1-null mice are neurologically normal with no impairment in the ability to learn, which may suggest that ERK2 compensates for the loss of ERK1 [62]. In contrast, it was shown that ERK2 knock-out mice are embryonically lethal [63].

In an adult mammalian central nervous system, ERK1/2 are expressed at a higher level in post-mitotic neurons. Immunohistochemical studies have demonstrated that ERK2 is localized in the soma and dendritic trees of neurons of the neocortex, the hippocampus, the striatum, and the cerebellum [64].

The ERK1/2 pathway mediates dopaminergic and glutamatergic signaling in the central nervous system and maintains normal activity of striatal neurons. Activation of ERK1/2 is important for associative learning, memory, visual cortical plasticity, etc. [65]. In addition, it is involved in the activation of D1 and D2 dopamine receptors in the striatum and ionotropic or metabotropic glutamatergic receptors in the dentate gyrus [66]. Moreover, it has been shown that the ERK1/2 signaling pathway plays an important role in the maintenance of spatial memory and long-term fear memory [67]. Another study has revealed the importance of ERK1/2 phosphorylation in neuronal development. Transiently repressed ERK1/2 phosphorylation in mice during the neonatal stage by intraperitoneal injection of MEK1/2 inhibitor, SL327, caused apoptosis of brain cells and had an effect on brain functioning: reduced LTP, impaired memory, and deficits in social behavior [68].

ERK1/2 signaling is involved in neuronal death, which is a major phenomenon in all neurodegenerative diseases including Parkinson’s disease [69]. Using the oligodendroglial CG4 cell line, it was shown that H_2_O_2_-induced cell death is prevented by the application of the ERK1⁄2 pathway inhibitor, PD98059 [70]. Additionally, it has been shown that nitric oxide produced by glial cells induces neuronal degeneration through ERK1⁄2 activation [71] and that this degeneration might be blocked by applying, PD98059 [72]. Application of another inhibitor of this pathway, U0126, also indicated that death of striatal neurons induced by dopamine was associated with ERK1⁄2 activation [73].

There are several processes that link ERK1/2 and Parkinson’s disease. In particular, these include: oxidative stress and mitochondria dysfunction, cell survival and apoptosis, neuroprotection, and inflammation. Regarding mitochondria dysfunction, it was found that, in the substantia nigra of PD patients, there is a mild deficiency in mitochondrial complex I [74]. Moreover, using confocal microscopy, it was established that phosphorylated ERK1/2 (p-ERK1/2) immune-reactivity was associated with mitochondrial proteins called MsSOPs and that some vesicular-appearing p-ERK1/2 granules enveloped enlarged mitochondria. In addition, p-ERK1/2 were found within the mitochondria of degenerating neurons derived from Parkinson’s disease patients and patients with Lewy body dementia [75]. There are also some other studies that support an idea that ERK1/2 inhibition activates both apoptotic and necrotic cell death-inducing pathways [76]. ERK1/2 directly phosphorylates mitochondrial transcription factor A (TFAM) on serine 117, which affects TFAM-DNA binding and, in consequence, leads to mitochondrial dysfunction. In addition, it was found that TFAM, which is downregulated by ERK1/2 in cells chronically treated with a complex 1 inhibitor, MPP+, regulates mitochondrial biogenesis [77]. Regarding oxidative stress, Wang et al. [78] have shown that the DJ-1 transcription factor interacted with ERK1/2 and was required for the nuclear translocation of ERK1/2. This translocation was suppressed in DJ-1 knock-down cells and DJ-1 null mice treated with an oxidative insult. Additionally, endoplasmic reticulum (ER) stress seems to play a critical role in the progression of Parkinson’s disease. Results obtained by Cai et al. [79] indicate that ER stress-induced apoptosis in PD might be inhibited by a basic fibroblast growth factor (bFGF). Administration of bFGF improved motor function recovery, increased tyrosine hydroxylase positive neuron survival, and upregulated the levels of neurotransmitters in the brain of a rat model of Parkinson’s disease. Another study has shown that a redox protein, thioredoxin-1, protects neurons from injuries and attenuates symptoms of Parkinson’s disease [80]. Additionally, it has been shown that the ERK1/2- and JNK1/2-c-Jun systems are linked with *L*-DOPA-induced neurotoxicity of dopaminergic neurons in a cellular model of PD [81] and that PI3K/Akt and ERK1/2 signaling pathways are involved in the protection of dopaminergic neurons against MPTP/MPP+-induced neurotoxicity [82]. In addition to that, it was found that, in LPS-induced PD models in vivo and in vitro, a flavonoid known as licochalcone A (Lico.A) significantly inhibited the production of pro-inflammatory mediators and microglial activation by blocking phosphorylation of ERK1/2 [83].

Lastly, it should be mentioned that some of the functions attributed to ERK1/2 in neuronal survival might be carried out by ERK5 since PD98059 and U0126 known as MEK1/2 inhibitors might inhibit the ERK5 pathway as well [84,85]. There is also a study that sheds light on the distinct roles of ERK1/2 and ERK5 in the survival of dopaminergic neurons under physiological conditions and acute oxidative stress. The latter condition is extensively linked to the molecular pathogenesis of Parkinson’s disease. The interaction between ERK5 and ERK1/2 pathways was found to promote basal survival of dopaminergic neurons when exposed to oxidative stress. When both pathways were inhibited, the decline in basal survival of MN9D dopaminergic cells after exposure to a toxic agent, 6-OHDA, was observed. In addition, it was found that ERK5 and ERK1/2 have different roles in neuronal metabolism. Activation of ERK5 promoted the survival of MN9D cells but had no influence on the toxic effect of 6-OHDA on these cells [86].

### 1.4. p38 MAPK Signaling 

The p38 MAPKs are strongly activated by extracellular stimuli such as UV light, heat shock, osmotic shock, inflammatory cytokines (e.g., TNF-α, IL-1β), or growth factors (e.g., CSF-1). Thus, these kinases are also known as stress-activated ones [87]. There are four isoforms of p38 MAPKs known as α, β, γ, and δ. All of them share up to 60% sequence similarities and 40% to 45% with other MAP kinase family members [88]. p38 MAPK isoforms have a different expression pattern. p38α MAPK is ubiquitously expressed in most cell types. p38β MAPK is mainly expressed in the brain while p38γ MAPK—in skeletal muscle and p38δ MAPK—is expressed in endocrine glands [89].

Regarding the role of p38α MAPK, it was found that the knockout of the gene encoding this protein is lethal [90] while mice lacking the *p38β* gene were viable and exhibited no apparent health problems. When embryonic fibroblasts from *p38β^−/−^* mice were analyzed, expression and activation of p38α MAPK, ERK1/2, and JNKs in response to cellular stress remained unchanged, which suggests that the α isoform of p38 MAPK is the main one responsible for controlling all of the detrimental consequences of the p38 MAPK activation such as microglia activation, neuro-inflammation, oxidative stress due to reactive oxygen species (ROS) accumulation, nitric oxide activity, and neuronal apoptosis [91,92,93].

It is worthy to note that several lines of evidence suggest that p38 MAPKs play a role in neuronal apoptosis, which is linked to Parkinson’s disease [94,95]. For instance, it has been reported that they induce apoptosis by phosphorylating Bcl-2 (B-cell lymphoma 2) family members [96]. Interestingly, phosphorylation of one such member, BimEL, on serine 65 may be a common regulatory point for cell death induced by both p38 MAPK and JNK pathways [97]. In addition, oxidative stress in dopaminergic neurons has been shown to trigger the p38 MAPK pathway which, in consequence, may lead to uncontrolled activation of apoptosis in cellular and animal models of Parkinson’s disease [98,99,100]. Together these data suggest that both oxidative stress and p38 MAPK operate to balance the pro-apoptotic and anti-apoptotic phenotypes of dopaminergic neurons. Some other studies show the link between the generation of ROS, initiation of the p38 MAPK/JNK signaling, and apoptosis of neuronal cells in different models of Parkinson’s disease [101,102,103,104,105]. Interestingly, it has been shown that the exacerbating effects of deletion of *Park2* gene (encoding parkin protein) on ethanol-induced ROS generation, mitophagy, mitochondrial dysfunction, and cell death were reduced by p38 MAPK inhibitor, SB203580, in vitro and in vivo. In the case of dopaminergic neurons it has been shown that deletion of this gene exacerbates ethanol-induced damage through p38 MAPK dependent inhibition of autophagy and mitochondrial function [106]. Similar data revealed that the p38 MAPK-parkin signaling pathway regulates mitochondrial homeostasis and neuronal degeneration in the A53T α-synuclein mutant model of Parkinson’s disease [107].

Attention to p38 MAPKs in terms of neurodegeneration is driven by the fact that these kinases are involved in dopaminergic signaling, which is a pathway known to be disrupted during Parkinson’s disease [93]. There is a study by Wu et al. [108] showing that degeneration of nigral dopaminergic neurons was accompanied by an increase in the level of p38 MAPKs and their phosphorylated forms. In agreement are the results published by Yoon et al. [109] showing that phosphorylation of p38 MAPKs by the LRRK2-ASK1 pathway regulated neuronal toxicity and apoptosis. Pharmacological inhibition of this kinase with SB203580 blunted MPTP neurotoxin induced cell apoptosis [110]. Similarly, neuronal protection was observed by applying another p38 MAPK inhibitor, SB239063 [111], or celastrol in rotenone-evoked neuroblastoma SH-SY5Y cellular model of Parkinson’s disease [112].

An important issue in Parkinson’s disease is neuro-inflammation that can be associated with alterations in glial cells including astrocytes and microglia. The response of neurons to activation of microglia promotes oxidative stress, inflammation, and cytokine-receptor-mediated apoptosis, which eventually contribute to the death of dopaminergic neurons and to the progression of the disease [113]. Rotenone, which is an inhibitor of the mitochondrial complex I, can directly activate microglial cells through the p38 MAPK pathway and initiate dopaminergic neuronal damage in substantia nigra, which ultimately results in parkinsonism. Unfortunately, the exact mechanism behind the selective degeneration of nigral dopaminergic neurons is not fully understood [103]. Moreover, it has been reported that montelukas, which is a cysteinyl leukotriene receptor antagonist, exerted neuroprotective effects in the rotenone-induced PD animal model through the attenuation of microglial cell activation and p38 MAPK expression [114]. It has been suggested that degeneration of nigral dopaminergic neurons was followed by an increase in the expression of p38 MAPKs, p53, and Bax (Bcl-2-associated X protein). Neurotoxins exhibited a similar effect on the level of these proteins in cultured pheochromocytoma PC12 cells, which shows that this phenomenon occurs both in vitro and in vivo. When activated, Bax is exported into the mitochondrial membrane where it oligomerizes and triggers mitochondrial apoptotic signaling. This observation strongly indicates that p38 MAPK/p53 stimulation of Bax can certainly contribute to rotenone’s neurotoxicity in models of Parkinson’s disease [108]. p38 MAPK also plays a role in neurotoxicity induced by MPTP [115].

Inflammation and autophagy are highly interdependent cellular processes. Autophagy plays an anti-inflammatory role and suppresses pro-inflammatory process by regulating innate immune signalling pathways and inflammasome activity [116]. Inflammatory signals also function to reciprocally control autophagy [117]. However, the mechanism of mutual regulation of both processes is not yet explained. A recent study has shown that the α isoform of p38 MAPK plays a direct and essential role in relieving autophagic control in response to an inflammatory signal by direct phosphorylation of UNC51-like kinase-1, which is the serine/threonine kinase involved in the autophagic cascade in microglia. Moreover, phosphorylation of UNC51-like kinase-1 by p38α MAPK inhibited activity of this kinase, disrupted its interaction with autophagy-related protein 13, ATG13, and, thus, reduced the level of autophagy [118]. Because autophagy disorders are more commonly associated with neurodegenerative diseases, the role of p38 MAPKs in autophagy was studied in a human neuroblastoma SK-N-SH cellular model of Parkinson’s disease. The studies revealed that microRNA (miR)-181a regulated apoptosis and autophagy by inhibiting the p38 MAPK/JNK pathway [119].

## 2. Conclusions

**Figure 2:**
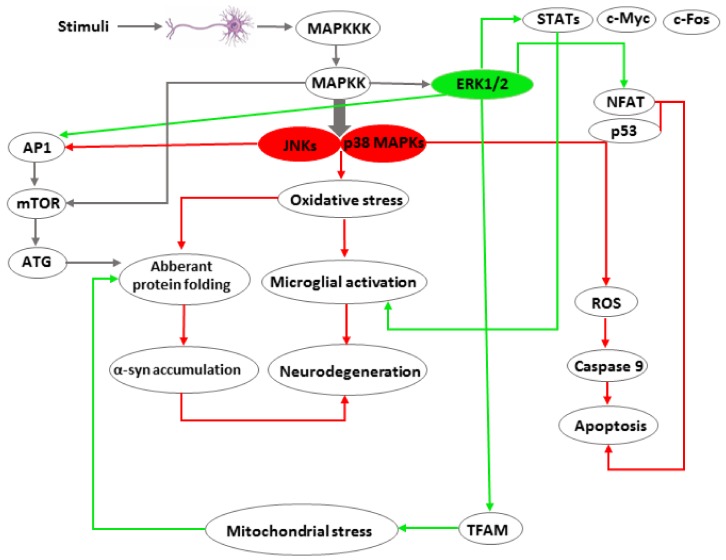
Involvement of MAPKs in processes that lead to Parkinson’s disease pathology. Different stimuli (stress stimuli, growth factors, cytokines, mitogens, pathogens, toxins) induce activation of MAPK pathways including activation of MAPKKK and MAPKK followed by phosphorylation of downstream targets such as JNKs, p38 MAPKs, and ERK1/2. JNKs and p38 MAPKs are grouped together due to their involvement in the “death pathway” (marked in red) while ERK1/2 is believed to promote cell growth and differentiation (marked in green). Activation of JNKs and p38 MAPKs promote oxidative stress and apoptosis, which are main contributors to PD pathogenesis. Oxidative stress may also cause microglial activation and chronic inflammation, which are toxic for brain cells and leads to PD pathology. In addition, ERK1/2 contribute to apoptosis of brain cells through the activation of NFAT and p53 and to neuronal inflammation through the activation of STATs. Both ERK1/2 and JNKs activate mTOR signaling, which promotes neurodegeneration such as that observed in PD.

Consistent with the critical role of MAPKs in key cellular activities including cell proliferation, differentiation, and survival or death. The MAPK signaling pathways have been implicated in the pathogenesis of many human diseases. Various observations suggest that they contribute to Parkinson’s disease-related pathological processes such as oxidative stress, neuro-inflammation, autophagy, and neuronal death (Figure 2). In addition, MAPK inhibitors demonstrate important neuroprotective properties upstream of the execution of apoptosis in dopaminergic neurons. Therefore, discovering the relationship between MAPK pathways and the prominent pathological processes observed during Parkinson’s disease progression may not only aid us to understand the etiology of this disease but also lend insight into molecular targets for the development of therapeutic drugs.

## Abbreviations

AMPA	α-amino-3-hydroxy-5-methyl-4-isoxazolepropionic acid (agonist of the AMPA receptor)
ASK1	Apoptosis signal-regulating kinase 1
AKT	protein kinase B
ATG13	autophagy-related protein 13
bFGF	basic fibroblast growth factor
Cdc42	cell division control protein 42 homolog
CNS	central nervous system
CSF-1	colony stimulating factor 1
ERK1/2	extracellular regulated kinase 1 and 2
EGFR	epidermal growth factor receptor
GPCR	G protein-coupled receptors
HMGB1	high mobility group protein 1
IL-1β	interleukin 1β
JIP1	JNK interacting protein 1
JNK	c-Jun N-terminal kinase
LBs	Lewy bodies
L-DOPA	*L*-3,4-dihydroxyphenylalanine
LRRK2	leucine-rich repeat kinase 2
LTD	long-term depression
MAP kinase	mitogen activated protein kinase
MEK3 and 6	mitogen-activated protein kinase kinase 3 and 6
MEKK1–3	mitogen-activated protein kinase kinase kinase 1 to 3
MLK2/3	mixed lineage kinases 2/3
MN9D	cell line used as a model of dopaminergic neurons
MNK	MAPK interacting protein kinase
MPTP	1-methyl-4-phenyl-1,2,3,6-tetrahydropyridine
MPP+	1-methyl-4-phenylpyridinium
MSK	mitogen and stress activated protein kinase
NGF	neurite growth factor
NMDA	*N*-Methyl-d-aspartic acid
NO	nitric oxide
6-OHDA	6-hydroxydopamine
PD	Parkinson’s disease
LTP	long-term potentiation
TH	tyrosine hydroxylase
Rac1	GTPase, member of the Rho family
ROS	reactive oxygen species
RTKs	receptor tyrosine kinases
RSK	ribosomal S6 kinase
SN	substantia nigra
STAT	signal transducer and activator of transcription
TAK1	transforming growth factor beta-activated kinase 1
TAO1/2	thousand and one amino acid kinases 1/2
TFAM	mitochondrial transcription factor A
TNF-α	tumor necrosis factor α
Tpl2	tumor progression locus 2 kinase
UNC51-like kinase-1	serine/threonine kinase involved in the autophagic cascade
NFAT	nuclear factor of activated T-cells
PI3K	Phosphatidylinositol-4,5-bisphosphate 3-kinase
